# Ciliary neurotrophic factor promotes motor reinnervation of the musculocutaneous nerve in an experimental model of end-to-side neurorrhaphy

**DOI:** 10.1186/1471-2202-12-58

**Published:** 2011-06-22

**Authors:** Petr Dubový, Otakar Raška, Ilona Klusáková, Lubomír Stejskal, Pavel Čelakovský, Pavel Haninec

**Affiliations:** 1 Department of Anatomy, Division of Neuroanatomy, Faculty of Medicine, and Central European Institute of Technology (CEITEC), Masaryk University, Kamenice 3, CZ-625 00 Brno, Czech Republic; 2 Department of Neurosurgery, 3rd Faculty of Medicine, Charles University, Prague, Czech Republic

## Abstract

**Background:**

It is difficult to repair nerve if proximal stump is unavailable or autogenous nerve grafts are insufficient for reconstructing extensive nerve damage. Therefore, alternative methods have been developed, including lateral anastomosis based on axons' ability to send out collateral sprouts into denervated nerve. The different capacity of a sensory or motor axon to send a sprout is controversial and may be controlled by cytokines and/or neurotrophic factors like ciliary neurotrophic factor (CNTF). The aim of the present study was to quantitatively assess collateral sprouts sent out by intact motor and sensory axons in the end-to-side neurorrhaphy model following intrathecal administration of CNTF in comparison with phosphate buffered saline (vehiculum) and Cerebrolysin.

The distal stump of rat transected musculocutaneous nerve (MCN) was attached in an end-to-side fashion with ulnar nerve. CNTF, Cerebrolysin and vehiculum were administered intrathecally for 2 weeks, and all animals were allowed to survive for 2 months from operation. Numbers of spinal motor and dorsal root ganglia neurons were estimated following their retrograde labeling by Fluoro-Ruby and Fluoro-Emerald applied to ulnar and musculocutaneous nerve, respectively. Reinnervation of biceps brachii muscles was assessed by electromyography, behavioral test, and diameter and myelin sheath thickness of regenerated axons.

**Results:**

Vehiculum or Cerebrolysin administration resulted in significantly higher numbers of myelinated axons regenerated into the MCN stumps compared with CNTF treatment. By contrast, the mean diameter of the myelinated axons and their myelin sheath thickness in the cases of Cerebrolysin- or CNTF-treated animals were larger than were those for rats treated with vehiculum. CNTF treatment significantly increased the percentage of motoneurons contributing to reinnervation of the MCN stumps (to 17.1%) when compared with vehiculum or Cerebrolysin treatments (at 9.9 or 9.6%, respectively). Reduced numbers of myelinated axons and simultaneously increased numbers of motoneurons contributing to reinnervation of the MCN improved functional reinnervation of the biceps brachii muscle after CNTF treatment.

**Conclusion:**

The present experimental study confirms end-to-side neurorrhaphy as an alternative method for reconstructing severed peripheral nerves. CNTF promotes motor reinnervation of the MCN stump after its end-to-side neurorrhaphy with ulnar nerve and improves functional recovery of the biceps brachii muscle.

## Background

Microsurgical reconstruction of interrupted nerve is generally based on end-to-end neurorrhaphy of stumps without tension. To overcome more extensive defects of peripheral nerves, autologous grafts prepared from cutaneous nerves are usually used [1-3]. It is difficult to repair nerve, however, if proximal stump is not available or autogenous nerve grafts are insufficient for reconstructing extensive nerve damage. Alternative methods have therefore been developed, including the use of conduits [4] and lateral anastomosis (end-to-side neurorrhaphy), first mentioned in 1903 [5]. End-to-side neurorrhaphy was revived by experiments that confirmed an ability of axons to send out collateral sprouts into denervated nerve stump [6-9]. The evidence of predominance in collateral sprouting by sensory or motor axons remains controversial. There are reports about collateral sprouting primarily involving sensory axons with only minimal growth into motor branches [10,11], while others describe good motor reinnervation [6,12]. A similar capacity of both intact sensory and motor axons to send collateral sprouts into a denervated nerve stump was demonstrated in our previous experiments [13].

Clinical application of end-to-side neurorrhaphy is not common, however, owing to a lack of sufficient experimental results. In addition, only a few papers are known relating to support of collateral sprouting following end-to-side anastomosis of peripheral nerves. It is postulated that both afferent and motor axon sprouting may be controlled by cytokines and/or neurotrophic factors. Candidate factors that may stimulate axonal sprouting include CNTF [14].

Ciliary neurotrophic factor (CNTF) is a naturally occurring protein initially identified by its ability to support the survival of parasympathetic neurons of chick ciliary ganglion in vitro [15] and subsequently purified from sciatic nerves [16,17]. CNTF is a member of the interleukin-6 family of cytokines including interleukin-6, CNTF, leukemia inhibitory factor, cardiotrophin-1, oncostatin M, and interleukin-11 [18]. CNTF is now known to be specifically expressed by astrocytes and Schwann cells [18-20] and enhances the survival of sensory and motor neurons [21-25]. Cerebrolysin (Ebewe Pharma, Austria), a peptidergic nootropic drug, is a solution containing free amino acids and biologically active peptides showing neurotrophic and neuroprotective effects revealed by in vivo and in vitro experiments [26-29]. In addition, Cerebrolysin enhances axonal sprouting of neurons in culture [30].

Although CNTF has been shown to promote the survival of neurons, little is known regarding its efficacy in the adult end-to-side neurorrhaphy model. Therefore, we utilized our model for end-to-side anastomosis of the distal musculocutaneous nerve (MCN) stump with intact ulnar nerve (UN) [9,13] to test the effect of CNTF on the formation of collateral sprouts and functional reinnervation of the biceps brachii muscle in comparison to Cerebrolysin.

## Results

### Quantitative analysis of labeled DRG and spinal motor neurons

The pool of labeled neurons for the UN of intact rats averaged 3086 ± 238 dorsal root ganglion (DRG) and 291 ± 52 motor neurons (Additional file 1, Table S1). The pool of retrogradely labeled neurons of rats operated for end-to-side neurorrhaphy was detected by distinct red, green and yellow (mixed) fluorescence on longitudinal sections through both the spinal cord segments (C6-Th1) and DRG of the same levels. The numbers of DRG and spinal motor neurons labeled by red, green or yellow fluorescence were assessed and are summarized in Additional file 1, Table S1.

**Figure 1:**
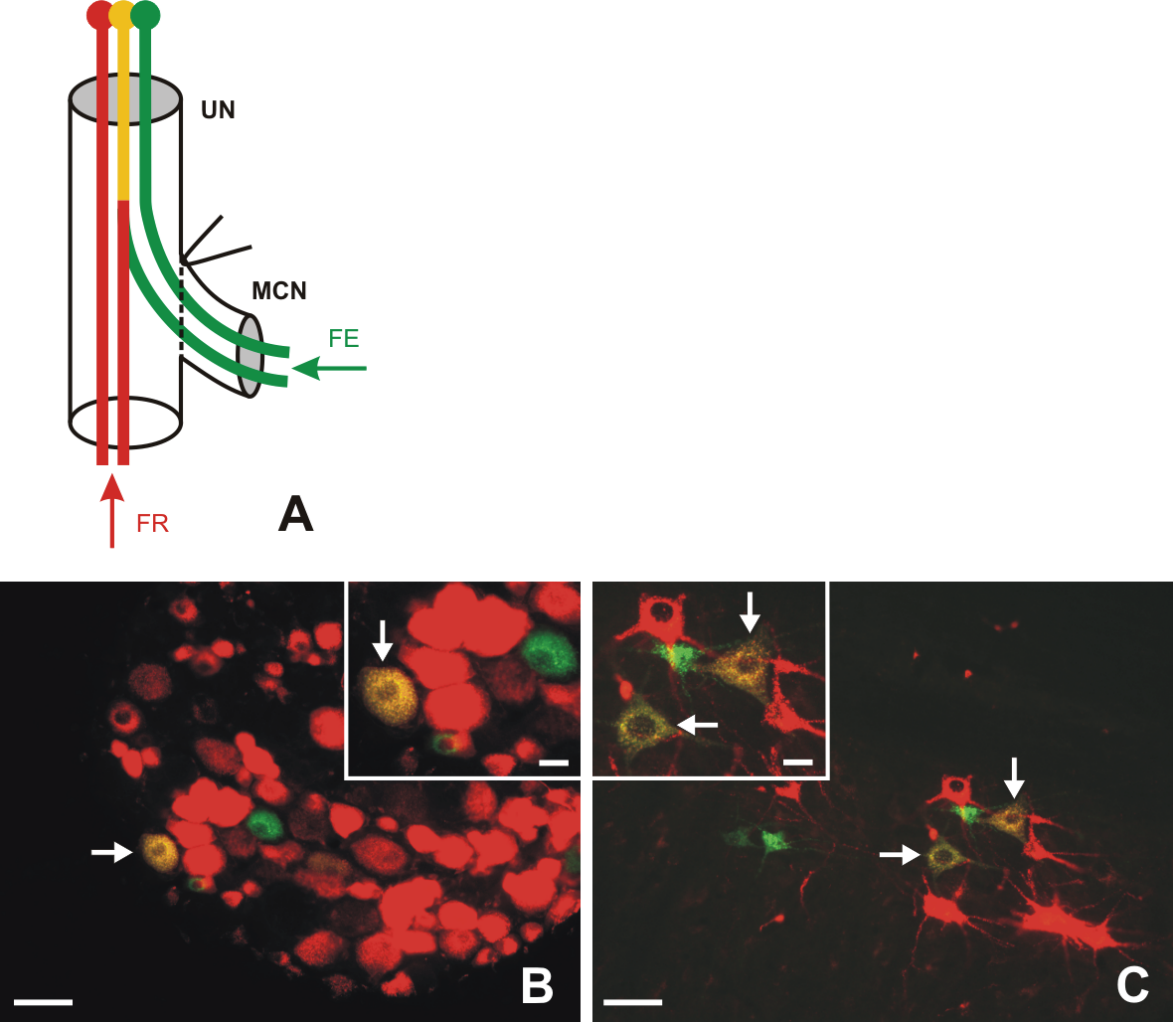
**A Schematic drawing of Double retrograde labeling of neurons**. A schematic drawing of double retrograde labeling of neurons following end-to-side neurorrhaphy of MCN stump with UN. FE - Fluoro-Emerald, FR - Fluoro-Ruby. B, C - Representative sections of C8 dorsal root ganglion and spinal cord segment. Representative cryostat sections of C8 dorsal root ganglion (B) and C8 spinal cord segment (C) illustrating retrograde labeled neurons. Arrows indicate double- (yellow-) labeled neurons of UN sending collateral sprouts into the distal stump of MCN. Scale bars = 100 μm; 40 μm for insets.

The largest numbers of motor and DRG neurons were labeled by red fluorescence, corresponding with accumulation of retrogradely transported Fluoro-Ruby (Figure 1b, c). These neurons are associated with donor motor and sensory axons present in the UN. The green fluorescence indicated neurons whose axons were damaged during surgical treatment and regenerated only into the MCN distal stump. Similar proportions of green-labeled motor and DRG neurons were found in vehiculum- and Cerebrolysin-treated rats. In contrast, CNTF treatment induced approximately a twofold increased proportion of green-labeled motoneurons while the proportion of labeled sensory perikarya remained similar to that in vehiculum- and Cerebrolysin-treated rats.

A yellow color in labeled neurons resulted from a mixture of red and green fluorescence and indicated those neurons the donor axons of which were present in the UN and had sent out collateral sprouts into the distal stump of MCN. Double-labeled (yellow) neurons were detected in both the spinal ventral horn and DRG (C6-Th1) of the operated side. The ratio of double-labeled to total labeled motor neurons was very similar whether following intrathecal administration of vehiculum or Cerebrolysin, but it was significantly higher after CNTF delivery. In contrast, the ratio of double-labeled to total labeled DRG neurons was very similar in all experimental groups following intrathecal administration of vehiculum, Cerebrolysin, as well as CNTF. This means that intrathecal application of vehiculum and Cerebrolysin resulted in similar ratios of double-labeled to all labeled motor and DRG neurons. A significantly higher proportion of double-labeled motor neurons in comparison to DRG neurons was found 2 months after intrathecal application of CNTF (Additional file 1, Table S1).

The proportions of motor neurons directly regenerating axons (green) or those sending off collateral sprouts from UN motor axons (yellow) were approximately doubled in CNTF rats when compared with Cerebrolysin treatment. This is illustrated also by the summary percentage of labeled motor neurons contributing to reinnervation of MCN stumps (see, Table 1), which was 17.1% for CNTF in comparison with 9.9 and 9.6% for vehiculum and Cerebrolysin, respectively.

**Table 1:** A comparison of the number of myelinated axons and labeled DRG and motor neurons contributing to MCN

MCN	Number of	Number of labeled sensory	Number of labeled	% of motor neurons
	myelinated axons	and motor neurons	motor neurons	to MCN
	(n ± SD)	to MCN (n ± SD)	to MCN (n ± SD)	
**Intact**	1259 ± 98	1857 ± 115	308 ± 41	16.6
**PBS**	1473 ± 135‡	515 ± 40‡	51 ± 13‡	9.9
**Cerebrolysin**	1282 ± 124	477 ± 35‡	46 ± 11‡	9.6
**CNTF**	782 ± 78‡†	601 ± 34‡†	103 ± 20‡†	17.1

A comparison of the mean number of myelinated axons, all labeled neurons (DRG and motor neurons) and motor neurons contributing to MCN in the intact rats and the MCN stumps after end-to-side anastomosis in PBS-, Cerebrolysin- and CNTF-treated rats as well as percentage of motor neurons. Although total number of myelinated axons in MCN stump of rats treated with CNTF is lower than in those treated with PBS or Cerebrolysin, note that the number and percentage of motor neurons (labeled by green and yellow fluorescence) contributing their axons to MCN stump is significantly higher in CNTF-treated rats.

‡ indicates statistical significance (P < 0.05) when comparing rats of experimental groups with intact rats; † indicates statistical significance (P < 0.05) when comparing rats with ALZET minipumps filled with CNTF and Cerebrolysin or PBS.

Generally, Cerebrolysin like vehiculum did not support preferential collateral sprouting from motor or sensory axons of donor nerve in the model for end-to-side anastomosis of the UN and MCN. In contrast, intrathecal application of CNTF supported formation of collateral sprouts and direct regeneration much more by motor than by sensory axons of the donor UN.

### Behavioral test

CNTF treatment resulted in a significantly higher behavioral test score in comparison with vehiculum- or Cerebrolysin-treated rat groups (Table 2). Correlation of the behavioral test with morphometric features of regenerated axons is provided below.

**Table 2:** Number and diameter of myelinated axons, thickness of their myelin sheaths and score of behavioral test

MCN	Number of myelinated	Axonal diameter	Myelin sheath thickness	BT ± SD
	axons (n ± SD)	(μm ± SD)	(μm ± SD)	
**intact**	1259 ± 98	4.08 ± 2.11	1.37 ± 0.64	5.00 ± 0.00
**PBS**	1473 ± 135‡	1.79 ± 0.03‡	0.61 ± 0.01‡	3.53 ± 0.52‡
**Cerebrolysin**	1282 ± 124#	2.16 ± 0.03+‡	0.81 ± 0.01+‡#	4.08 ± 0.49‡
**CNTF**	782 ± 78+‡	2.11 ± 0.03+‡	0.79 ± 0.01+‡	5.00 ± 0.00+

Mean numbers of myelinated axons, their diameters and thickness of myelin sheaths in intact MCN and in MCN stumps after end-to-side anastomosis in phosphate buffered saline (PBS-), Cerebrolysin- and CNTF-treated rats as well as their mean scores for behavioral test (BT).

‡ indicates statistical significance (P < 0.05) when comparing rats of experimental groups with intact rats; + indicates statistical significance (P < 0.05) when comparing rats with ALZET minipumps filled with CNTF or Cerebrolysin and PBS; # indicates statistical significance (P < 0.05) when comparing rats with ALZET minipumps filled with Cerebrolysin and CNTF.

### A morphometric analysis of myelinated axons regenerated into the MCN

**Figure 2:**
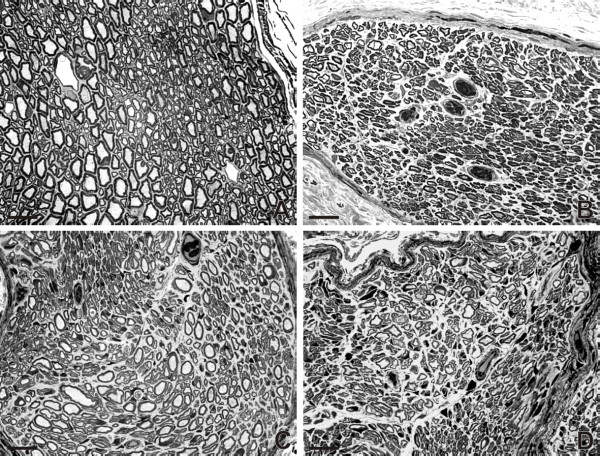
**Representative semithin sections through MCN**. Representative transverse semithin sections through intact MCN (A) and MCN stumps 2 months after their reconnection with the UN and intrathecal application of vehiculum (B), Cerebrolysin (C), and CNTF (D) for 2 weeks. Scale bars = 10 μm.

**Figure 3:**
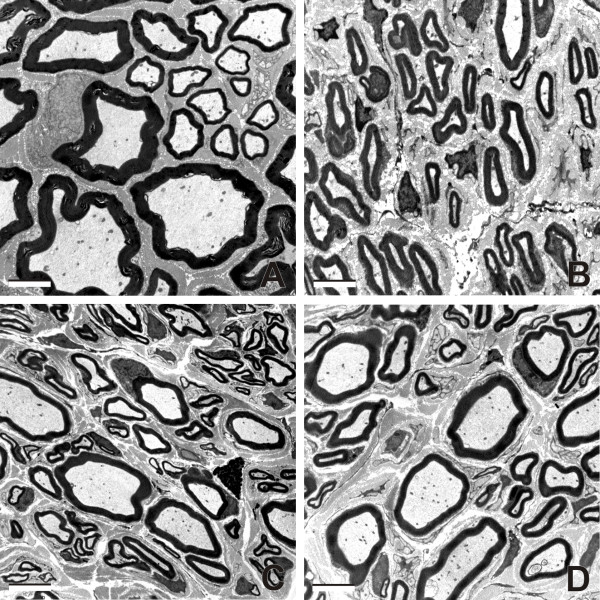
**Representative ultrathin sections through MCN**. Electron micrographs showing representative myelinated axons in cross sections through intact MCN (A), and MCN stumps 2 months after their reconnection with the UN and intrathecal application of vehiculum (B), Cerebrolysin (C) and CNTF (D). Scale bars = 2 μm.

Representative transverse sections through the intact MCN and MCN stumps 2 months after their reconnection with the UN by end-to-side anastomosis and treated with vehiculum, Cerebrolysin and CNTF are illustrated in Figure 2. Occurrence of the myelinated axons, their viability, and absence of the surviving myelin profiles in the MCN stumps was confirmed by electron microscopy (Figure 3). Intrathecal administration with vehiculum or Cerebrolysin for 2 weeks and survival for 2 months after surgery resulted in a significantly higher number of myelinated axons regenerated into MCN stump in comparison with CNTF treatment. However, mean diameter of the myelinated axons regenerated into the MCN stump and their myelin sheath thickness for Cerebrolysin- or CNTF-treated animals were larger than were those of rats treated with vehiculum. The mean axon diameter and thickness of myelin sheaths were similar for the groups of Cerebrolysin- and CNTF-treated rats, but mean scores for the behavioral (grooming) test were significantly the best for CNTF-treated animals. The diameter and myelin thickness of regenerated axons did not reach the values for myelinated axons in MCN removed from intact rats (Table 2).

CNTF-treated rats displayed significantly higher numbers of all labeled neurons as well as of motor neurons contributing to MCN stumps compared to vehiculum- and Cerebrolysin-treated animals while the number of myelinated axons was reduced (Table 1). The average number of myelinated axons per neuron (motor and sensory) was 1.3 for CNTF but 2.7 and 2.9, respectively, in the Cerebrolysin and vehiculum groups. Moreover, the percentage of motoneurons contributing to MCN stumps was higher for CNTF treatment than for vehiculum or Cerebrolysin treatment and was comparable with the MCN of intact rats. However, the proportion of green- and yellow-labeled sensory perikarya of DRG remained in the CNTF group similar to those in the vehiculum- and Cerebrolysin-treated rats. The results suggest that a higher proportion of motor axons was regenerated into the MCN stump following CNTF treatment, although the total number of myelinated axons was lower than in the vehiculum- and Cerebrolysin-treated rats (Table 1).

### Electromyography (EMG)

No EMG denervation elements in measured muscle (fibrillations, positive waves) were found in animals of any group. Due to the general anesthesia, evaluation of voluntary muscular activity was impossible. The state of reinnervation was assessed with regard to motor responses following UN stimulation. Motor response was elicited in all measured muscles.

Amplitudes of responses assessed as degree of restored innervation of muscle fibers [31] were significantly higher in the biceps brachii muscles of Cerebrolysin- and CNTF-treated rats when compared with vehiculum-treated rats (Table 3). In contrast, amplitudes measured in the flexor carpi ulnaris muscles were similar in all experimental groups of rats. This corresponds with the comparable number of neurons labeled with red and green fluorescence in all experimental rats (Additional file 1, Table S1) and indicates a similar range of injury to a small number of UN axons during surgical treatment.

**Table 3:** EMG motor responses in the biceps brachii and flexor carpi ulnaris muscles

	BB mean amplitude	FCU mean amplitude
	(mV ± SD)	(mV ± SD)
**PBS**	4.8 ± 2.8	17.8 ± 2.1
**Cerebrolysin**	14.8 ± 4.4*	16.2 ± 4.7
**CNTF**	12.9 ± 3.3*	16.7 ± 1.8

Mean amplitudes of EMG motor responses in the biceps brachii (BB) and flexor carpi ulnaris (FCU) muscles 2 months after end-to-side neurorrhaphy of MCN stump with UN and intrathecal application for 2 weeks of PBS, Cerebrolysin or CNTF.

* indicates statistical significance (P < 0.05) when comparing rats with ALZET minipumps filled with CNTF or Cerebrolysin and PBS.

No differences were found in latencies among experimental animal groups, indicating that the remyelination of axons [32] was advanced in all rats at the time of measurement.

## Discussion

Despite improvements over the last decade in surgical techniques for the brachial plexus, surgical outcome and functional restoration of the affected arm is still very limited [33]. Especially difficult is the treatment of patients with avulsion of cervical roots, because direct reconstruction by neurotization techniques is impossible and the results of reimplanting avulsed roots have been controversial and presented for only a small number of patients [34].

Therefore, alternative methods are being developed that including lateral anastomosis, which was first reported in 1903 [5] and revived by experiments that confirmed an ability of axons to send out collateral sprouts into denervated nerve stump [6-8]. A double-labeling method using different fluorescent tracers has been applied to obtain morphological evidence of collateral reinnervation following end-to-side neurorrhaphy [35,36]. Morphological evidence of collateral sprouts sent by intact sensory and motor axons of the UN has also been obtained by retrograde labeling of the neurons using one type of molecule (dextran) conjugated with two different fluorophores. This approach to neuronal labeling based on one type of molecule (e.g., Fluoro-Ruby, Fluoro-Emerald) is suitable for quantitative morphological evaluation of collateral sprouting [9,13].

Stimulation of collateral sprouts into donor nerve can arise when a balance of factors inhibiting and stimulating axonal growth is disrupted along the intact (donor) nerve fibers. The factors consist of mechanical and molecular barriers of the myelin sheaths and endoneurial extracellular matrix [37-39]. It is well known from in vitro studies that a great number of molecular factors contribute to collateral sprouting, but their origins remain mostly unknown. Although the effects of various factors promoting nerve regeneration are well documented for many different models of peripheral nerve injury, there exists only a limited number of references concerning the factors that stimulate collateral sprouting [15,40].

Generally, most factors probably arise from Schwann cells which lost contact with axons during Wallerian degeneration. These reactive Schwann cells and their processes are also able to promote axonal growth [11,41]. Thus, the distal stumps of the severed nerve containing reactive Schwann cells produce several factors that promote axonal growth [42]. Some of these, such as CNTF, basic fibroblast growth factor-2, and insulin-like growth factors I and II, are able to enhance collateral sprouting [14,43-46]. Therefore, applications of axonal promoting molecules together with a meticulous surgical technique are believed to be key factors for axonal collateral sprouting after end-to-side neurorrhaphy.

CNTF prevents the cell death of axotomized sensory and motor neurons [21,23,47] and promotes axonal regeneration and sprouting of motor axons [14,23,48,49]. In addition, the presence of CNTF in the Schwann cells of both the proximal and distal nerve stumps of transected peripheral nerve is consistent with its acting as a sprout-inducing agent [17,23]. Schwann cells provide stimuli for inducing and/or maintaining collateral sprouting of certain axons, but they are probably not sufficient in the absence of other mechanisms [50].

### Numbers of labeled motor and DRG neurons

The fact that no significant differences were found in the proportions of double-labeled DRG or motor neurons in our experimental model following vehiculum or Cerebrolysin treatment indicates similar capacities of rat motor and sensory axons for collateral sprout formation. These results are in agreement with those obtained in rats operated for end-to-side neurorrhaphy without a treatment [13]. In comparison to Cerebrolysin and vehiculum treatments, our results revealed that intrathecal CNTF application induced a significantly higher proportion of double-stained motor versus DRG neurons - indicating an increased support of motor as opposed to DRG neurons to send out collateral sprouts.

The percentage of double-stained motor and primary sensory neurons was relatively low in all experimental groups (1.4-3.3%). This suggests that collateral sprouting from intact axons to the recipient nerve is not the sole mechanism of functional reinnervation. The neurons labeled by green fluorescence regenerated their axons directly into the MCN stump, indicating injury of a small amount of UN axons during preparation of the perineurial window. However, no EMG denervation elements (fibrillations, positive waves) were found in the flexor carpi ulnaris muscles of any experimental animals. The facts that there were no significant differences in either the proportions of green fluorescence-labeled DRG and motor neurons after application of vehiculum or Cerebrolysin or of DRG neurons in the CNTF experimental group suggest a similar impairment of their axons during surgical manipulation and stimulation of axon regeneration. However, a double proportion of green fluorescence-stained motor neurons in CNTF-treated rats suggested that CNTF promoted the growth of regenerated motor axons into the MCN stump. The motor axons directly regenerated into the MCN stump certainly contributed to the functional reinnervation of the muscles that is probably reflected in better results of the behavioral test and EMG measurement in comparison to that of vehiculum-treated rats. A presence of UN axons directly regenerated into MCN stump following end-to-side neurorrhaphy is very similar to the Oberlin procedure for reinnervation of the biceps brachii muscle using end-to-end neurorrhaphy of a part of the UN with the recipient MCN [51].

### Numbers of labeled neurons and myelinated axons regenerated into MCN stumps, and functional recovery

Thickness of myelin sheaths and calibers of axons in the MCN stump show that CNTF, like Cerebrolysin, distinctly promotes maturation of regenerated axons. Support for maturation of regenerated axons by intrathecal administration of Cerebrolysin was also observed in our previous experiments [29]. In contrast to Cerebrolysin treatment, CNTF application led to there occurring a lower number of myelinated axons in the MCN stump and simultaneously to a higher number of labeled motor neurons regenerating their axons into the MCN stump. In addition, behavioral tests were significantly improved. This suggests that CNTF promotes motor reinnervation of MCN stumps. These results accord with a finding that muscle contraction requires activation of relatively small proportions of the motor neurons supplying a given muscle [52]. Thus, the biceps muscle fibers reinnervated by a lower number of motor axons may provide better behavioral tests in CNTF than in Cerebrolysin rats despite lower numbers of myelinated axons in the MCN stumps.

It is generally accepted that insufficient functional recovery of muscle following nerve lesion is due mainly to incorrect and aberrant motor axon reinnervation [53-56]. First, the probability of regrowing axons' successfully navigating to correct structural targets is increased by many regenerating branches being sent off by individual proximal axons and running distal to nerve lesion. Each proximal axonal stump sends out up to 25 regenerating branches that grow in parallel through the distal nerve stump [57,58]. Second, persistence of excessive collateral branches in the endoneurial tubes of the distal nerve stump leads to polyinnervation of one muscle fiber [59,60], which is a poorer physiological condition for muscle's functional recovery [54,61-63].

In the case of our experiments, however, we must distinguish between branches of regenerating axons running parallel through distal stump of MCN and collateral sprouts sent off by axons of donor UN after end-to-side neurorrhaphy. The numbers of axons regenerated to MCN need not correspond with the numbers of labeled neurons [64,65]. When we compared the numbers of myelinated axons regenerated in MCN stumps and the number of labeled sensory and motor neurons contributing to innervations of MCN stump, this indicated that excessive numbers of collateral branches were being sent off by individual regenerating axons. This was mainly the case in vehiculum and Cerebrolysin treatment. Nevertheless, CNTF treatment significantly reduced the numbers of myelinated axons in MCN stumps and simultaneously increased the proportion of motor neurons the axons of which contributed to reinnervation of the MCN stumps. This was related also with improved behavioral tests. The results suggest that a higher proportion of motor axons was regenerated into the MCN stump following CNTF treatment.

**Figure 4:**
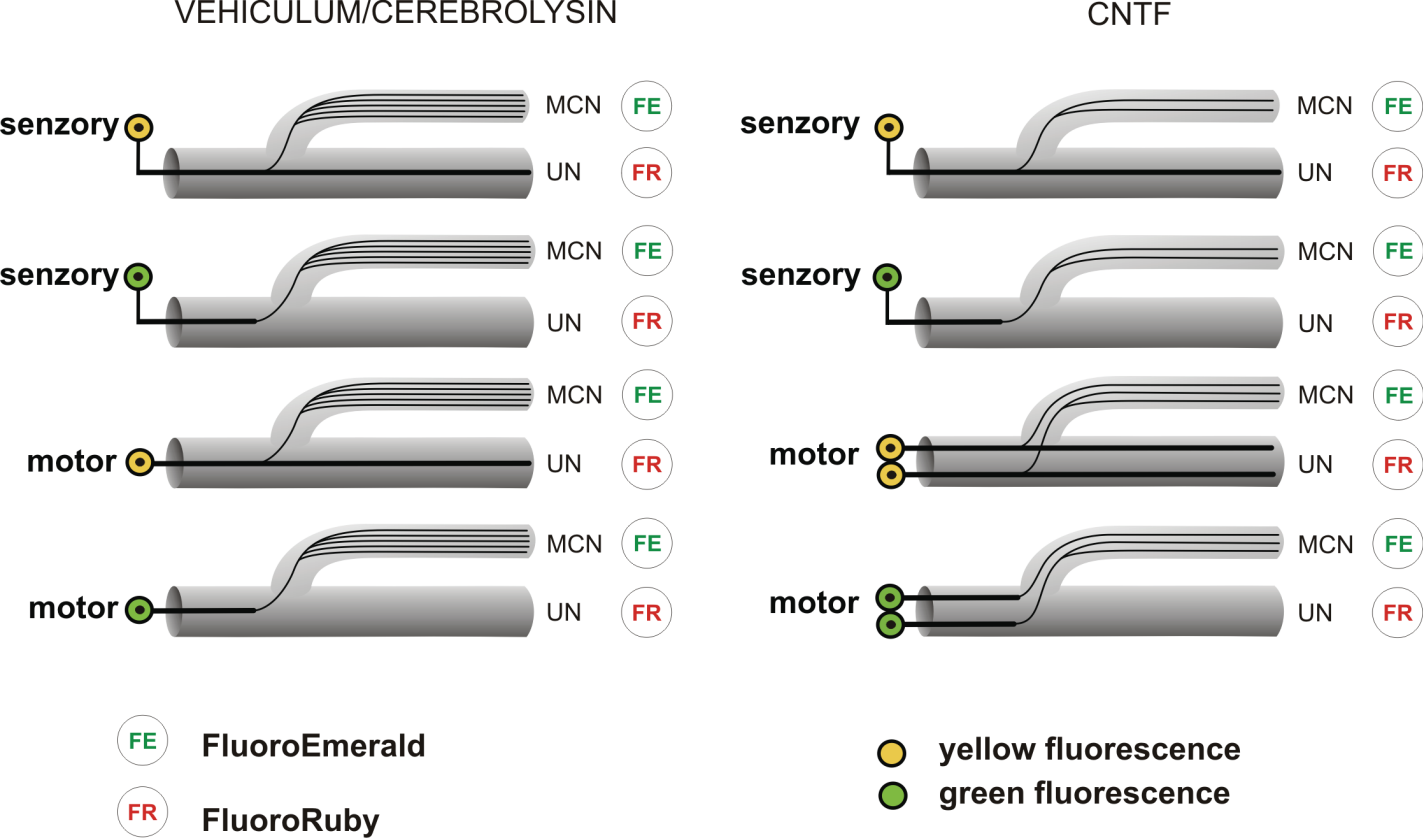
**Schematic drawing of a possible explanation of paradoxical differences between labeled neurons and numbers of myelinated axons**. Schematic drawing of a possible explanation of paradoxical differences between labeled neurons and numbers of myelinated axons in the MCN stump after its end-to-side neurorrhaphy and CNTF treatment when compared with vehiculum/Cerebrolysin application. CNTF-treated animals have shown approximately double the number of UN motor neurons that sent off lateral sprouts into (yellow fluorescence) or directly innervated (green fluorescence) the MCN stump and simultaneously reduced the numbers of sensory and motor myelinated axon branches.

The experiments with CNTF-treated animals revealed that exogenous CNTF stimulates nerve regeneration by promoting a greater number of elongating axons in the distal stump [48] and motor axon sprouting from nodes of Ranvier [14]. While both mechanisms may function during initial stages of end-to-side neurorrhaphy, pruning is commonly considered a mechanism for functional adaptation of regenerated axons to new connections over long periods of time. That a higher proportion of motor neurons regenerated their axons into the MCN stump while, at the same time, total numbers of myelinated axons decreased after CNTF treatment could be explained by pruning of sensory myelinated axons and redundant motor branches (Figure 4). It is known from the literature that exogenous CNTF has a beneficial effect on motor function recovery of the sciatic nerve after axotomy [66] and reduces denervation-induced atrophy of muscle [67]. Myelinating Schwann cells of intact peripheral nerve display a high level of CNTF protein and mRNA, but they are dramatically reduced in the distal segment of the injured sciatic nerve [23,68]. In contrast, CNTFRa mRNA levels increase in the distal nerve stump [69] and skeletal muscle after nerve injury [70]. While we can speculate that soluble CNTFRa, mainly in denervated muscle, could collaborate with exogenous CNTF to support preferential motor innervation by pruning of sensory myelinated axons and redundant motor branches (see also [56,71]), precise data on regulation of the pruning process by CNTF are lacking.

CNTF has pleiotropic effects on cells in the nervous system, the mechanisms of which are not entirely understood. Thus, there can be an alternate explanation for our finding that CNTF promotes motor reinnervation of the MCN stump after its end-to-side neurorrhaphy. In particular, a reduction in total numbers of myelinated axons in the MCN stump after CNTF application can be explained on the basis that CNTF potentiates axonal regeneration by promoting a greater number of elongating axons in the distal nerve stump [48], which thereby prevents collateral branching.

Surprisingly, some experimental results have shown that reduced axonal branching distal to nerve reconstruction does not always lead to improved functional recovery [54,62,72]. It seems that reduction of polyinnervation of motor end plates is an additional critical factor that may ameliorate functional muscle recovery [54,55]. While similar amplitudes of EMG muscular response among CNTF and Cerebrolysin groups indicated comparable amounts of reinnervated muscle units, decreased numbers of myelinated axons, increased proportion of motor neurons for MCN reinnervation and (especially) improved behavioral test in the CNTF rats may indicate changes in the pattern of motor end plate reinnervation of the biceps muscle fibers in contrast to that of Cerebrolysin-treated rats. Nevertheless, the proportions of mono- and polyinnervation of the motor end plates following end-to-side neurorrhaphy remain to be elucidated.

## Conclusions

In conclusion, the experimental results presented here confirm that end-to-side neurorrhaphy is an alternative method for reconstructing severed peripheral nerves. This is supported by our clinical experience, and especially when insufficient sources of motor fibers are available [73,74]. The experimental results revealed that both collateral sprouting from intact motor axons and direct motor reinnervation from partially injured donor nerve contribute to functional muscle reinnervation. Both types of motor reinnervation were significantly promoted by intrathecal CNTF administration in comparison with vehiculum and Cerebrolysin and improved functional recovery of the biceps brachii muscle following application of end-to-side neurorrhaphy.

## Methods

### Animals, surgical procedures and experimental groups

Twenty-four adult female rats (Wistar, Charles River, AnLab Brno, Czech Republic) weighing about 250 g were divided randomly into four groups: intact (n = 6), vehiculum (n = 6), Cerebrolysin (n = 6), and CNTF (n = 6). All surgical experiments and treatments were carried out according to the relevant European Communities Council directive and approved by the local ethics committee at the Faculty of Medicine, Masaryk University Brno, Czech Republic. The rats were housed in an animal facility at temperature 20°C and with a natural day-night cycle. Food and water were available ad libitum.

The experimental rats were anesthetized by i.p. injection (0.2 ml/100 g body weight) of a mixture of xylazine (4 mg/ml) and ketamine (40 mg/ml). The right MCN and UN were exposed at the level of the axillary fossa. The epineurium of the UN was dissected away in a small region and a minimal incision was made in the perineurium to create a perineurial window. The MCN was transected and its distal stump was attached in an end-to-side fashion using a 10/0 atraumatic monofilament polyamide (Ethilon) stitch in the epineurium. The proximal MCN stump was tightly ligated and turned back to prevent spontaneous reinnervation. The skin wound was closed with 5/0 sutures.

A brain infusion cannula (Brain Infusion Kit 3-5 mm, ALZA, Palo Alto, California, USA) was introduced into the right lateral ventricle after making a cut in the scalp and drilling an opening 1 mm rostral and 1 mm right of the coronal suture. The cannula was introduced to the depth of 3.8-4 mm and connected using a polyethylene catheter tube with an ALZET 2002 osmotic minipump (rate 0.5 μl/h, 2 weeks) implanted subcutaneously in the back of the rat. The osmotic minipumps of the vehiculum rat group were filled with a phosphate buffered saline (PBS) solution. The CNTF group animals were infused with recombinant rat CNTF (R&D Systems, Minneapolis) diluted in vehiculum for final concentration of 50 μg/ml, while the osmotic minipumps of the Cerebrolysin group of animals were filled with a commercially available and non-diluted Cerebrolysin (Ebewe Pharma, Austria). The infusion speed corresponded to 0.6 μg/day of CNTF and 12 μl/day of Cerebrolysin. The total time of vehiculum, Cerebrolysin and CNTF intrathecal administration was 2 weeks, and all animals were left to survive for 2 months.

### Behavioral analysis

Behavioral analysis of active elbow flexion in the right forelimb was evaluated and scored in the cages using the grooming test [75] 2 months after surgery. Statistical comparison of behavioral data was by analysis of variance followed by appropriate post hoc tests (Tukey's and Dunnett's multiple comparisons) using Statistica 9.0 software (StatSoft, Inc.). Statistical significance was accepted at the 5% level (p < 0.05).

### Electromyography

EMG measurement was performed 2 months after surgery. A Medelec Synergy 2-channel instrument (Viasys Healthcare, Old Woking, Surrey, England) was used for the EMG investigation. The motor responses were recorded through disposable monopolar 12 mm needle electrodes 0.4 mm in diameter from the biceps brachii and flexor carpi ulnaris muscles that are exclusively innervated by the UN [76]. The reference needle electrode was inserted into the contralateral axilla and the ground electrode into the tail. A surface bipolar electrode was used for stimulation of the musculocutaneous nerve. The stimulation site was situated 2 mm proximally to the point of end-to-side suture. The distance from the stimulation site to the end of the recording needle electrode was 15 ± 2 mm in the biceps brachii muscle and 25 ± 2 mm in the flexor carpi ulnaris. Duration of the rectangular stimulus was 0.02 ms. Supramaximal stimulating intensity was reached at 1-6 mA. Each measurement was repeated to ensure consistency. Amplitude, area and latency of the motor response were measured at 22°C and correlated. The data were statistically evaluated by Mann-Whitney U test (Statistica 9.0; StatSoft, Inc.) with p < 0.05 as the level of significant differences between the tested values.

### Retrograde tracing

Double retrograde labeling was used to study the origin of the nerve fibers which had regenerated from the UN into the end-to-side attached distal stump of the MCN. After behavioral test and EMG measurement, the animals were re-anesthetized (see above) and the UN and distal stump of MCN were re-exposed and cut about 10 mm distal to their end-to-side attachment. The stumps of UN and MCN were inserted into yellow pipette tips filled with 10 ml of 10% Fluoro-Ruby or Fluoro-Emerald (Molecular Probes, Inc.), respectively (Figure 1a). The stumps were gently washed with PBS for 20 minutes following the tracer exposure, and the wound was closed with 5/0 sutures. The UN of intact rats was cut and labeled with 10% Fluoro-Ruby like the nerve of operated animals.

The animals were left to survive for the next 4 days, then deeply anesthetized (using pentobarbital) and perfused with PBS followed by Zamboni's fixative [77]. The C6-C8 spinal cord segments and corresponding DRG were removed and immersed in Zamboni's fixative overnight. The tissue samples were washed in 10% and 20% sucrose overnight. Serial longitudinal cryostat sections (20 mm) were taken from the spinal cord segments and the DRG, collected onto chrome-alum coated slides, then mounted into VectaShield medium (Vector Laboratories, Burlingame, CA, USA). The sections were viewed and digitalized in a Leica DMLB fluorescence microscope using a G/R filter to estimate single- and double-labeled neurons. Only labeled motor and DRG neurons with distinct nucleoli were counted.

To verify differences, the Mann-Whitney U test was run using Statistica 9.0 software (StatSoft, Inc.) with p < 0.05 as the level of significant differences between the tested values.

### Morphometric evaluation of myelinated axons in the MCN

Segments removed from intact and operated MCN were fixed overnight by immersion in the fixative solution containing 4% depolymerized paraformaldehyde, 1.5% glutaraldehyde, and 10% sucrose in cacodylate buffer (0.1 M, pH 7.2). The samples were next post-fixed in 1% osmium tetroxide after washing in cacodylate buffer (0.1 M, pH 7.2) and then embedded into Durcupan (Durcupan ACM, Fluka) by the standard procedure. The transverse semithin sections, 80 nm thick, were stained with toluidine blue. Six randomly selected sections were digitalized under a Leica DMBL light microscope equipped with a DFC-480 digital camera at final magnification of 600×.

The total number and diameter of myelinated axons as well as the myelin sheath thickness were counted and measured by means of a Lucia-G (Laboratory Imaging, Prague, Czech Republic) computer-assisted image analysis system from pictures digitalized in BMP format.

Diameter of axons and their myelin sheath thickness were computed from cross-section areas that provide the highest precision, greatest accuracy and least bias [78,79]. At least 500 myelinated axons cut perpendicularly were measured for each MCN of the control and each experimental group with elimination of spurious and oblique profiles.

The total number of myelinated axons, axon diameters, and myelin thickness were compared among the MCN of intact and operated rats supported by intrathecal administration for 2 weeks of vehiculum, Cerebrolysin and CNTF. The data were statistically evaluated by one-way analysis of variance with post hoc comparisons of means using Statistica 9.0 software (StatSoft Inc.). Statistical significance was accepted at the 5% level (p < 0.05).

In order to illustrate the myelin axon regeneration in the MCN stumps, the ultrathin sections were cut using an Ultra-cut 701701 ultramicrotome (Leica Microsystems, Wetzlar, Germany), stained with lead citrate and uranyl acetate, then examined using a Morgagni 268 electron microscope (FEI Company, Hillsboro, OR, USA).

## Competing interests

The authors declare that they have no competing interests.

## Authors' contributions

PD conceived, designed and coordinated the study and wrote the manuscript. PH conceived, designed and coordinated the study and carried out the experiments. OR, IK, LS and PČ participated in acquiring and analyzing the presented data. All authors gave final approval of the version to be published.

## Supplementary Material

Additional file 1 **Table S1 - Quantitative analysis of labeled spinal motoneurons and DRG neurons**. Quantitative analysis of labeled spinal motoneurons and DRG neurons of intact rats (n = 6) and rats following UN-MCN end-to-side anastomosis and intrathecal treatment with PBS, Cerebrolysin and CNTF. Red-labeled perikarya belong to neurons with single axons present in the UN; green-labeled perikarya belong to neurons whose single axons were damaged during surgical treatment and regenerated only into the MCN distal stump; yellow-labeled perikarya resulted from a mixture of red and green fluorescence and indicate those UN neurons whose axons gave off an additional daughter branch that adjoined and grew into the distal stump of MCN. † indicates statistical significance (P < 0.05) when comparing rats with ALZET minipumps filled with CNTF and Cerebrolysin or PBS. Click here for file
